# Synthesis of libraries and multi-site mutagenesis using a PCR-derived, dU-containing template

**DOI:** 10.1093/synbio/ysaa030

**Published:** 2021-01-05

**Authors:** Gretchen Meinke, Nahide Dalda, Benjamin S Brigham, Andrew Bohm

**Affiliations:** Department of Developmental, Molecular and Chemical Biology, Tufts University School of Medicine, Boston, MA 02111, USA; Department of Developmental, Molecular and Chemical Biology, Tufts University School of Medicine, Boston, MA 02111, USA; Department of Developmental, Molecular and Chemical Biology, Tufts University School of Medicine, Boston, MA 02111, USA; Department of Developmental, Molecular and Chemical Biology, Tufts University School of Medicine, Boston, MA 02111, USA

**Keywords:** protein engineering, DNA library synthesis, multisite mutagenesis, directed evolution, antibody design

## Abstract

Directed DNA libraries are useful because they focus genetic diversity in the most important regions within a sequence. Ideally, all sequences in such libraries should appear with the same frequency and there should be no significant background from the starting sequence. These properties maximize the number of different sequences that can be screened. Described herein is a method termed SLUPT (Synthesis of Libraries via a dU-containing PCR-derived Template) for generating highly targeted DNA libraries and/or multi-site mutations wherein the altered bases may be widely distributed within a target sequence. This method is highly efficient and modular. Moreover, multiple distinct sites, each with one or more base changes, can be altered in a single reaction. There is very low background from the starting sequence, and SLUPT libraries have similar representation of each base at the positions selected for variation. The SLUPT method utilizes a single-stranded dU-containing DNA template that is made by polymerase chain reaction (PCR). Synthesis of the template in this way is significantly easier than has been described earlier. A series of oligonucleotide primers that are homologous to the template and encode the desired genetic diversity are extended and ligated in a single reaction to form the mutated product sequence or library. After selective inactivation of the template, only the product library is amplified. There are no restrictions on the spacing of the mutagenic primers except that they cannot overlap.

## Background

Error-prone PCR and other completely random mutagenesis schemes are highly inefficient methods for identifying mutations that enhance or alter protein function. Simple probability calculations show that such screens are heavily biased toward amino acids with codons that differ by one or two bases compared to the starting sequence, and the natural degeneracy of the genetic code dictates that some amino acids are six times more likely than others to be sampled. Moreover, stop signals (encoded by three different codons) are statistically more likely than the 10 amino acids that are encoded by just one or two different codons. More importantly, random screening ignores the vast and ever-growing database of sequence and structural information that might inform the search for well-folded proteins with enhanced or altered activity (1).

While unbiased mutagenesis can facilitate the discovery of mutations that would have been overlooked by more rational approaches, it is often the case that the critical mutations identified through random screening cluster in regions that could have been predicted through sequence and/or structure-based analysis (2). Residues at or near an active site, those close to a biologically important interface, or those involved in functionally important movements are more likely than others to modulate activity. These and other ‘data-driven engineering’ principles have been successfully used in many cases to improve enzyme activity, enhance stability and/or alter specificity (3). In short, while it is usually very difficult to predict precisely which mutation(s) will yield a desired effect, it is often relatively easy to identify the regions within a protein sequence where the likelihood of finding favorable mutations is high. Indeed, in recent years a variety of software tools have been developed to help identify the regions where mutations are likely to be most fruitful (3–6).

The vastness of sequence-space is always a major factor in screening projects. With 19 alternate amino acids at each position, there are 1900 possible single-site mutations of a 100 amino acid protein. Such a protein has over 2.5 million two-site variants and over 2 × 10^16^ five-site variants. Even highly efficient bacterial screens sample far fewer sequences in each selection cycle. As a consequence, the overall sampling tends to be exceptionally sparse, and many promising variants are likely missed. Rationally designed libraries, wherein the genetic variation is concentrated at specific regions help circumvent many of these issues. The physical construction of such libraries, however, can present a bottleneck in the protein engineering process. This is especially the case if one wishes to achieve relatively uniform sampling of the desired sequence-space and there are multiple and widely spaced regions within the sequence that one wishes to vary.

The problem of synthesizing targeted DNA libraries has been approached in a variety of ways. Many methods rely on some form of gene assembly where a series of overlapping fragments are assembled to form the final product (7). In the ‘Assembly of Designed Oligonucleotides (ADO)’ approach (8) synthetic overlapping oligonucleotides with variable regions are designed so that there are single-stranded gaps in the assembly in the regions that are being varied. Thus, a polymerase used to fill these gaps generates the complementary strand in the variable regions. In this case, the product is formed in a single reaction, but oligos covering the entire gene are required. Another set of recently reported approaches, termed ‘Ligation of Fragment Ends After PCR’ (LFEAP) and ‘Assembly of Fragment Ends After PCR (AFEAP)’ involves two PCR cycles per mutation and results in PCR products with overhangs at each end which self-assemble to form the final product (9, 10). This utilizes fewer primers than ADO but is still not ideal because two PCR reactions are required for every fragment in the gene assembly. New England Biolab’s HiFi Assembly and other ‘Gibson assembly’ approaches are conceptually similar but require just one set of primers for each mutation. In this case, the overhangs required for assembly are generated using an exonuclease (11). Though these approaches require fewer oligos than ADO and the individual fragments can be prepared in parallel, the overall complexity of the procedure (a series of separate PCR reactions, each with specific primers for each mutated region, followed by assembly and usually ligation to form the final product) is still somewhat involved. This is also the case for megaprimer and overlapping extension-based approaches including OSCARR (12). In these approaches, the products of intermediate rounds of PCR are used as primers for subsequent PCR cycles(13). Thus, most approaches for making widely spaced multisite libraries require either multiple PCR cycles to generate intermediate products that are later assembled or a single reaction with a collection of primers that spans the entire gene. While these approaches have been successfully used in a variety of large-scale mutagenesis and protein engineering efforts (2), they are relatively cumbersome, and the intermediate PCR steps make it difficult to control the precise distribution of randomized nucleotides at the mutated sites. As a consequence, some sequences may be oversampled, while others may be completely absent from the resulting library.

Two important alternatives to the methods discussed above are the Quikchange Multi Site-Directed Mutagenesis approach developed by Invitrogen and an adaption of the Kunkel mutagenesis approach described by Caucheteur *et al.* (14, 15). The Quikchange approach has many of the same advantages as presented here, but the nontemplate strand can compete with the mutagenic primers. Also, as discussed later, the DNA melting step prior to primer annealing most likely enhances primer competition for the template and hinders a uniform distribution of bases in the product strands. The second approach relies on M13 phage to generate a single-stranded template and a *dut ung Escherichia coli* strain which occasionally incorporates the RNA base uracil into this template. As in the method described here, genetic variation is introduced via primers that contain degenerate bases. These primers are extended and ligated to form the product strand.

Herein, we present a method named SLUPT (Synthesis of Libraries via deoxyuridine (dU)-containing PCR Templates) for quickly constructing highly targeted DNA libraries with mutated regions that may be close or far from one another in the DNA sequence. SLUPT can also be used to efficiently make multiple, simultaneous, specific substitutions within a target sequence. This method is largely similar to that described by Kunkel and Caucheteur *et al.* (14, 15), but the single-stranded template is made by PCR. This simplifies the process of template preparation considerably. SLUPT also uses a higher fidelity polymerase that lacks exonuclease activity (Phusion U in place of T4 polymerase). As with the Kunkel approach, the starting sequence is almost completely absent from SLUPT products, and the method allows multiple regions to be altered in a single reaction using just one primer for each region that is modified. Moreover, when SLUPT is used to synthesize targeted DNA libraries, alternative nucleotides at the varied positions are stoichiometrically well balanced. Thus, SLUPT is ideally suited for protein engineering efforts where having all of the sequences within the library at the same concentration maximizes the number of protein variants that can be effectively screened.

## Methods

### Library generation and mutagenesis via SLUPT

#### Part 1: Preparation of dU-containing ssDNA template

##### Synthesis and purification of the dU-containing template

The wt template for the recombinase studies is 1050 bp in length. This sequence was amplified using dU-containing NTP mixtures (GeneAmp, N8080270) using either Taq DNA polymerase (New England Biolabs, M0267S) or Phusion-U Hotstart polymerase (ThermoFisher Scientific, F555S), following the manufacturer’s protocol. For this step, the forward 5′ primer must be 5′ phosphorylated, the 3′ reverse primer is not. All primers in this study were synthesized at the smallest scale possible (IDT DNA), with standard desalting and no other purification. No special effort was made to ensure that the stoichiometry of bases at degenerate positions within the ordered nucleotides was exactly balanced (we relied on the DNA synthesis company for this). dU-containing PCR product DNA was gel extracted using various kits (Machery-Nagel, New England Biolabs, Zymo) with similar efficiency. The dU-containing PCR reaction was typically repeated using the purified PCR product as template. The second PCR step affords an opportunity for scale-up at this stage by performing multiple PCR reactions (e.g. ten 50 µl reactions). These second PCR reactions are normally cleaned up via spin columns, but gel extraction is recommended if there are multiple bands. For this 1 kb template, ten 50 µl PCR reactions yielded ∼ 20 µg of dU-containing dsDNA.

##### Digestion of the 5′phosphorylated ‘top strand’ with Lambda exonuclease

Typically, 2 μg of the purified, dU-containing PCR product is digested with Lambda exonuclease, enough for many subsequent reactions. The reaction contained the dU-PCR product, 4 μl of 10× lambda exonuclease buffer, 10 U lambda exonuclease (New England Biolabs, M0262S), and water to 40 μl. The reaction was incubated at 37°C for 1.5 h, followed by heat inactivation at 75°C for 10 min. Typically, we perform multiple 40 µl reactions (i.e. 5–10 reactions) for scale-up. The ssDNA was extracted from an agarose gel made using SYBR Green II RNA gel stain (Invitrogen, S7564) for better visualization of ssDNA. Recovery of ssDNA from gel slice is typically performed using a DNA gel extraction kit (Machery-Nagel, 740609.50). The concentration of the ssDNA was calculated using the standard extinction coefficient of 33 µg/ml and the length of the ssDNA. The ssDNA transient template was stored at −20°C until ready for use. This should be enough for hundreds of SLUPT reactions.

##### Testing the ssDNA template

The quality of the ssDNA was assessed by performing standard 25 µ; PCR reactions using Taq DNA polymerase either with or without prior treatment with Antarctic Thermolabile Uracil DNA glycosylase (UDG) (New England Biolabs, M0372S). For this test, a series 10-fold dilutions of the ssDNA is used as the template: none, 1:10, 1:100, 1:1000, 1:10 000. For each dilution, a 10 µl ± UDG reaction which contains 1 µl 10× UDG reaction buffer, 1 µl ssDNA, ± 1 µl UDG and water to 10 µl was prepared. The reaction was incubated for 30′ at 37°C. Next, standard PCR was performed using primers for the start and end of the gene for all the dilutions using 1–2 µl template. No PCR product in the presence of UDG indicates no template contamination. PCR product in the absence of UDG indicates how low a dilution may be used for the next steps.

#### Part 2: Annealing, extension, ligation and amplification of the product DNA

##### Design of donor primers

The donor primers should be designed such that their annealing temperature (excluding the mutated region) is above 55°C, and they should contain 15–20 bases on each side of the desired mutated region that are complementary to the template sequence. All donor primers were ordered with a 5′ phosphate, as this is needed for the ligation step. No special purification other than standard desalting was requested. We have used a primer as short as 29 bp, with a single nucleotide change near the center and 10 and 18 homologous bases on either side, respectively. The longest primer tested to date is 68 bp in length, with multiple mutation regions in the center, flanked by 20 and 21 homologous bases, respectively. There is significant flexibility in the primer design but using very short or very long primers may require empirical testing. All successful donor primers used in this study are shown in the Supplementary Table S1. Lyophilized donor primers were resuspended in 10 mM Tris pH 8.5 or sterile milliQ water, typically at a 100 µM concentration, and stored at −20°C.

##### Annealing, extension and ligation of the primers

Typically donor primer: ssDNA ratios around 1000:1 work well; lower ratios will also work, but as the primer: template ratio decreases there is an increased likelihood of skipping a primer and obtaining the template sequence instead of the desired variants. The amount of ssDNA template used here depends in part on the previous UDG test. Typically, for the recombinase study, we used ssDNA template at a concentration of ∼2.5 ng/µl or ∼10 fmol/µl. In this step, annealing occurs at room temperature, which favors random annealing of the primer mixtures to the template. Typically 10 µl annealing reactions are performed in PCR tubes. Each reaction contains 1 µl 10× Taq ligase buffer, 10 fmol ssDNA, 10 pmol of 5′ PCR forward primer, 10 pmol of donor primer mixture, and water to 10 µl total. Incubate at room temperature for 30′. For the extension and ligation reaction, in a PCR tube, place 1 µl of the annealed sample, 1 µl 10× Taq DNA ligase buffer, dNTP mixture for a final concentration of 100 µM, 2.5 units Taq DNA ligase (New England Biolabs, M0208S), 0.75 units Phusion-U-Hotstart polymerase, water to 10 µl. Incubate at 55°C for 30′.

##### Inactivation of the template strand

Digest each reaction with UDG for 30′ at 37°C. For example, in a 10 µl UDG digestion reaction, use 2.5 µl gap filled template, 1 µl 10× reaction buffer, 1 µl UDG and water to 10 µl.

##### Amplify the single-stranded library or mutant via PCR

Use 2.5 µl UDG-digested sample as template in a 50 µl PCR reaction with forward and reverse primers. No special conditions are necessary. Using Phusion polymerase (New England Biolabs, M0530) (which does not tolerate dU in the template) will further ensure that none of the template sequence remains in the double-stranded product, though this is not usually a problem. PCR clean-up is performed using Machery-Nagel kits.

A step by step protocol of the SLUPT method will be deposited to Protocol Exchange (https://protocolexchange.researchsquare.com/).

##### Sanger sequencing of SLUPT PCR products

The sequence of all libraries and mutations were characterized by Sanger sequencing performed either by the Tufts University Core Facility or by Genewiz. DNA sequences and traces were analyzed using SnapGene software (from Insightful Science; available at https://www.snapgene.com/).

##### Molecular graphics

Molecular graphics figures were prepared using PyMOL (The PyMOL Molecular Graphics System, Version 2.3.4, Schrödinger, LLC).

##### Cloning, transformation and NGS analysis

One microgram of PCR product library and 2 µg of empty pEVO plasmid (18) were separately digested in 15 µl reactions with BsrgI and XbaI (both from New England Biolabs). After gel purification and cleanup using a Macherey-Nagel kit, the library was ligated into the vector in a 50 µl reaction containing 20 µl of plasmid (13 ng/µl), 15 µl of insert (22 ng/µl), 10 µl 5× ligase buffer and 5 µl of T4 DNA ligase (Invitrogen, 15224041). For the transformation, the entire reaction was added to 1 ml of homemade rubidium chloride competent Top 10 cells. After a 1 h incubation on ice, the cells were heat shocked for 90 s at 42°C and then incubated on ice for 2 min. They were then grown at 37°C for 1 h before an aliquot was removed for plating on chloramphenicol and subsequent colony counting. The remaining cells were transferred to a 65 ml flask of LB and grown overnight in the presence of chloramphenicol before plasmid purification. The purified plasmids were digested with BsrgI and Xba1, and the SLUPT DNA library was gel purified. The DNA library was then fragmented by sonication and subjected to paired end 150 bp sequencing using an Illumina MiSeq instrument at the Tufts Genomics Core facility. The resulting reads were aligned to the parent sequence using Bowtie2 (bowtie-bio.sourceforge.net/bowtie2/index.shtml), and the resulting BAM file was visually inspected using IGV (software.broadinstitute.org/software/igv). The statistics presented in Table 2 were calculated using a short python script that used the pysam pileup function (pysam.readthedocs.io/en/latest/index.html). The output of this script includes the base position, the fraction of reads with each of the four bases and the total number of reads contributing to the count at each position. This program output is included in the Supplementary materials. We noticed that the error rate is larger at both ends of the PhiX control alignment and at the 5′ end of the library alignment. We believe these increased errors are an artifact of the sequencing. The average error rates and standard deviations on these error rates are reported without the 25 bases at the 5′ end of the library and without 5 bases at either end of the PhiX genome, which was spiked into the library as an internal control.

##### Anti-CTLA4 scFv Antibody SLUPT library generation

A plasmid containing the anti-CTLA4 scFv antibody was obtained from Addgene (#85436). A scFv fragment was generated by standard PCR for use as the template in this study. An anti-CTLA4 scFv library was then created as described above, using the mutagenic donor primers presented in Figure 4 and Supplementary Table S1.

##### MSCS python script

The MSCS script is designed to help users select degenerate codon mixtures that encode a desired set of amino acids. Based on user input, the script generates a sorted list of the 3375 possible codon mixtures that can be easily synthesized and wherein the mixed bases are at same concentrations. The script is written in python3, and it requires the biopython module. This module is freely available at https://biopython.org/ and can be installed on many Linux systems by issuing the command apt-get install python3-biopython. To run the script from the command line type python3 MSCS.py. Users will be prompted for a list of amino acids they would like encoded and then a list of weights (−1.0 to 1.0) for each of these amino acids. (Negative weights indicate that the user prefers not to see the respective amino acid near the top of the output.) Users are also prompted for penalty parameters for missing amino acids, for encoded, but not requested amino acids, and for stop codons. The default parameters generally work well, but users are encouraged to experiment with other values and see the effect these have on the sorted output. Base mixtures are indicated using the standard code: B = C/G/T, D = A/G/T, H = A/C/T, K = G/T, M = A/C, N = A/C/G/T, R = A/G, S = C/G, V = A/C/G, W = A/T, Y = C/T.

The pEVO plasmid used to amplify both the test library and the five-primer mutagenesis test is based on a pBAD plasmid sequence from Addgene, and is covered by a Materials Transfer Agreement.

## Results

**Figure 1.**
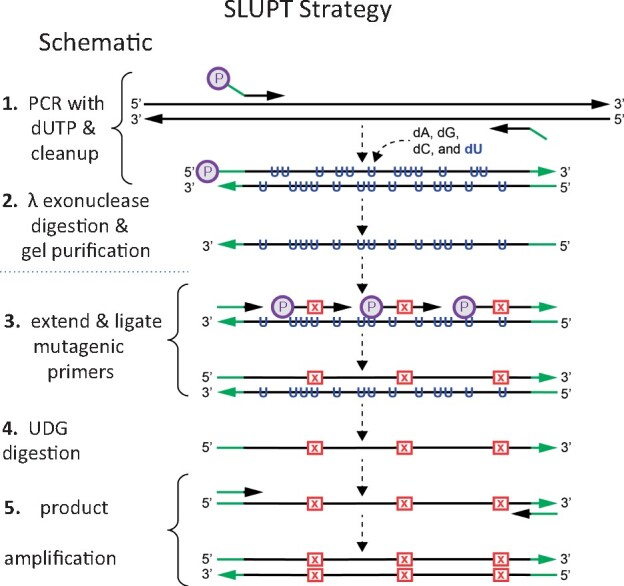
Schematic overview of the SLUPT strategy. Step 1: The gene of interest is amplified with a 5′ phosphorylated top strand primer and dNTP’s containing dU (blue). The primer for the bottom strand is not phosphorylated. Optional, nonhomologous regions (e.g. to introduce restriction enzyme sites) are shown in green. Step 2: The phosphorylated strand is selectively degraded by lambda exonuclease to create the uracil-containing single stranded template. Step 3: An end-primer complementary to the 3′ terminus and 5′ phosphorylated internal primers containing altered bases are annealed to the uracil containing single strand template. Altered bases depicted as X’s in red box. Gap filling and ligation are performed by Phusion-U and Taq ligase to create a mutated, complementary strand. Step 4: The Uracil-containing single stranded template is digested by UDG. Step 5: The single-stranded product is made double stranded and amplified by PCR.

To illustrate the utility of this method, we describe below the synthesis and sequencing of libraries involving two well-known protein engineering targets, Cre recombinase and a single-chain antibody against CTLA4. We also show how SLUPT can be used to generate a series of Cre-based mutations. The SLUPT strategy, outlined in Figure 1, relies on a single stranded dU-containing template which is enzymatically inactivated before the final library is amplified. The single-stranded template is synthesized in a PCR reaction in which the primer for the top strand is phosphorylated and that for the bottom strand is not. An exonuclease is used to selectively degrade the top strand leaving the bottom, single-stranded template. Primers homologous to the 5′ end of the template and to the internal regions of the sequence which will be altered are then annealed to the template. After the primers are extended and ligated, the dU-containing template strand is inactivated, and the resulting single-stranded library is then made double-stranded and amplified by conventional PCR.

In practice the SLUPT protocol can be divided into two parts, template preparation and DNA synthesis. A single template preparation (Steps 1 and 2 in Figure 1) is sufficient for hundreds of subsequent library and/or mutagenesis reactions. The library is synthesized and selectively amplified in the second part of the procedure (Steps 3–5, Figure 1). This part can be completed in one afternoon.

### Recombinase library generation

**Figure 2.**
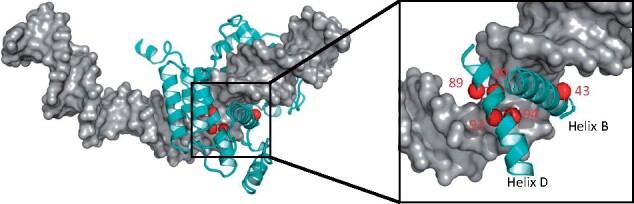
Structure-based library design of a Cre-based recombinase. (Left) A structural model of a Cre variant (cyan) bound to its target DNA (surface representation in gray) with only one monomer of the Cre tetramer shown. The location of the desired mutations is indicated by red spheres located at the C-alpha coordinates. (Right) Close-up of the helix B (with amino acid 43) and helix D (with amino acids 89, 90, 93, and 94).

**Figure 3.**
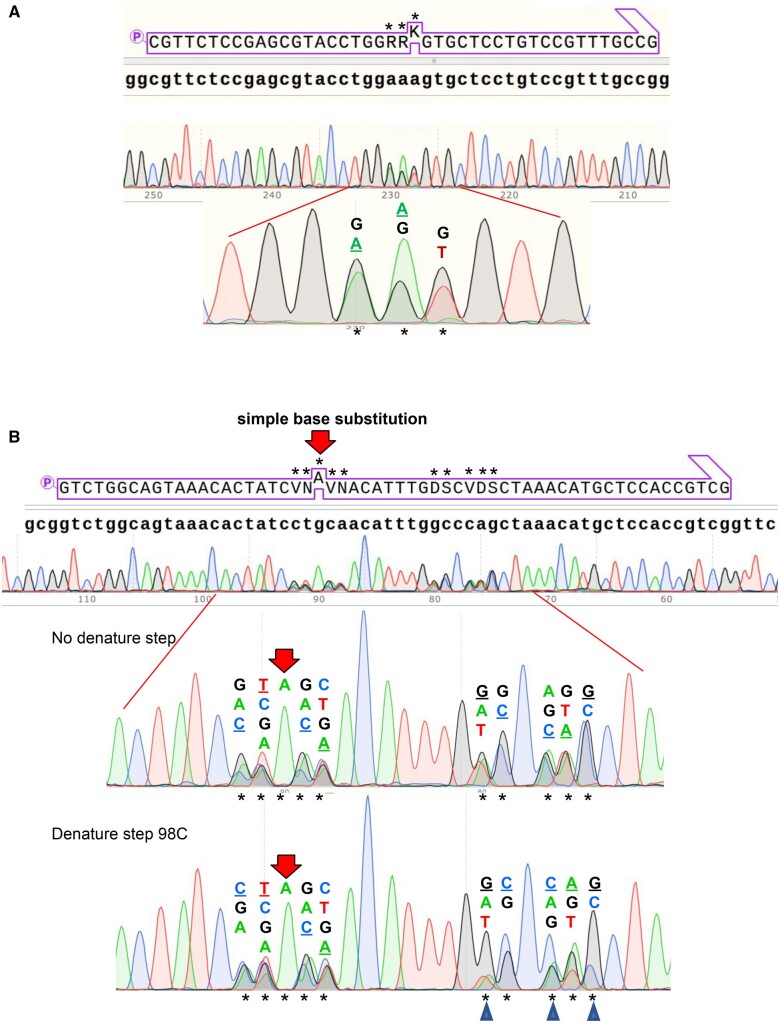
Sample sequencing results of the amplified library wherein two mutated regions are separated by ∼130 bp. Sanger sequencing chromatograms of the library in the mutated helix B region (**A**) and the mutated helix D region (**B**) of the recombinase. The 5′ phosphorylated donor primer is shown in a purple box above the starting sequence (lowercase bold). The location of the mutations within the primer are indicated by a *. An expanded view of the mutated region is shown, and the mutated bases are listed in order of their approximate peak height and the bases are colored according to their corresponding trace. Underlined bases are those that correspond to the starting sequence. The red arrow in B denotes a simple G > A base substitution. The library shown in C is identical to that shown in B, but the primers and template were heated to 95°C and then allowed to cool before the elongation/ligation step. Positions where the templated base is strongly favored are marked with blue triangles.

As an initial test, and to illustrate the utility of this approach, we generated a library of Cre recombinase variants with differences at amino acid positions 43, 89, 90, 93 and 94. These amino acids are in Helix B and Helix D of the enzyme, and both regions are shown to interact with the DNA substrate in recombinase crystal structures (Figure 2) (16). To limit the size of our library, we chose to include only a subset of the 20 amino acids at each of the 5 amino acids positions that were varied. The primers used are shown in Figure 3, and the amino acid changes are shown in Table 1.

**Table 1.** SLUPT library design for recombinase library 1

AA	Codon alteration^a^	Amino acid variants	AA frequency^b^	Rationale
**K43**	AAA > RRK	EDGKNSR	11211111	Charged, polar, gly
**L89**	CTG > VNA	AEGIKLQPRTV	11111111211	varied
**Q90**	CAA > VNA	AEGIKLQPRTV	11111111211	varied
**A93**	GCC > DSC	CASTG	11211	Small, u n charged
**Q94**	CAG > VDS	SKGQDVIRHMLEN	112112131121	varied

a Standard single letter codes for base mixtures are listed in this column. R = A/G, K = G/T, V = A/C/G, N = A/T/G/C, D = A/G/T and S = G/C.

b The AA (amino acid) frequency refers to the number of times the amino acid in the variants list is represented by the given codon alteration, respectively.

**Table 2.** Summary of NGS results at the altered nucleotide positions

Region 1		Region 2		Region 3		Region 4	
112 T > B	**T 44%**, G 33%, C 23%, a 0.1%	257 A > V	G 40%, **A 34%**, C 26%, t 0.1%	523 T > R	G 63%, A 36%, **t 0.4%**, c 0.0%	844 A > C	C 99%, **A 0.7%**, t 0.1%, g 0.0%
113 C > M	A 58%, **C 42%**, g 0.1%, t 0.0%	258 G > W	T 51%, A 48%, **g 0.1%**, c 0.1%	524 C > V	G 43%, **C 29%**, A 27%, t 0.1%	845 G > R	**G 59%**, A 41%, c 0.1%, t 0.0%
114 C > A	A 99%, **C 1.1%**, g 0.1%, t 0.0%	268 C > W	A 50%, T 49%, **c 0.1%**, g 0.1%	525 C > T	T 99%, **C 0.5 %**, g 0.1%, a 0.1%		
118 C > V	A 37%, G 34%, **C 29%,** t 0.0%	269 A > V	G 41%, **A 39%**, C 19%, t 0.1%				
119 G > V	**G 43%**, A 29%, C 27%, t 0.1%						
127 A > V	G 42%, **A 37%**, C 20%, t 0.1%						
128 A > R	**A 53%**, G 47%, T 0.1% c 0.0%						
130 G > V	**G 52%**, A 29%, C 19%,t 0.1%						
131 T > D	**T 46%**, A 28%, G 26%, c 0.1%						
132 G > K	**G 62%**, T 38%, a 0.1%, c 0.1%						

For each of the four altered regions, the first column indicates the nucleotide position and nature of the mutation, and the second column summarizes the NGS results. Bases in lower case were not encoded by the mutagenic primers and were not expected in the library. Bases in bold are encoded by the template. These data are taken from a much larger table that includes the fraction of A, T, G and C at each position of the sequence and has additional significant figures for all values (see pileup_output.xlsx in Supplementary materials).

A python script (included in the Supplementary materials and discussed in greater detail below) was used to select the specific variants at each amino acid position we modified. Our choices resulted in a hypothetical, targeted library encoding 124 416 different codons and 55 055 different protein sequences. As noted in Table 1, some of the degenerate codon mixtures encoded certain amino acids more than once. This is difficult to avoid when using simple base mixtures but can be avoided if more complex primer mixtures are utilized (17).

The degree of variation in the SLUPT generated library was visualized by Sanger sequencing. The synthetic oligonucleotides used to form the library should have equal amounts of the two, three or four bases we selected at each position selected for variation. As shown by the chromatographic sequencing traces in Figure 3A and B, the amounts of the four bases are in general agreement with the expected values. SLUPT can also be used to make simple base substitutions, as is the case at the position marked with a red arrow in Figure 3B, where Guanosine is mutated to Adenosine. The efficiency of the approach is highlighted by the absence of alternative bases at this position. Importantly, the starting sequence is not preferred, as might be expected if the U-containing template were not completely deactivated or if oligonucleotides with greater base complementarity selectively hybridized to the template.

Interestingly, the roughly equal distribution of bases at the selected, degenerate positions is much less evident if an initial 95°C denaturing step is performed when the donor primers are first added to the template. Inclusion of this heating step favors annealing of oligonucleotides that are most homologous to the starting sequence. This unequal distribution of bases is particularly evident when mutating a G/C base pair adjacent to another G/C pair that is not mutated (blue triangles in Figure 3C). Most likely, this is because the heating facilitates free exchange and competition between primers while the sample cools. This pattern is highly reproducible, and we believe the variation results from differences in the stability of different partially duplex structures. While the denaturing step in the annealing process should generally be avoided when making libraries, the reproducibility of the effect suggests that it may be useful in evaluating the relative energies of various mismatched duplex structures. It is notable that the absence of a denaturation step prior to primer annealing further differentiates SLUPT from procedures that involve PCR with mutagenic primers. These alternative procedures include multisite QuikChange, megaprimer extension and gene assembly schemes.

To better understand the diversity and quality of libraries synthesized via SLUPT, and to help evaluate the degree of library diversity after the SLUPT PCR product libraries are cloned into a plasmid and transformed into cells, we synthesized a second Cre-based library wherein 19 selected base pairs in four distinct regions were simultaneously altered. We used one mutagenic primer for each of the four altered regions (Supplementary Figure S1). To help evaluate the diversity of this library in bacterial cells, the library was cloned into a plasmid, and transformed into *E. coli*, yielding approximately 900 000 colony forming units. We then extracted the plasmids from an overnight bacterial culture, cut the library back out of the plasmids, and gel purified the excised DNA. The DNA encoding the Cre variant library was then submitted to the Tufts Genomics Core where it was fragmented by sonication and subjected to paired-end next generation sequencing (NGS). The same sample was submitted for Sanger sequencing and the resulting sequencing chromatograms are presented in Supplementary Figure S1. Although the Sanger chromatograms are obviously a far less accurate measure of variable base incorporation and background, we found general agreement between the Sanger sequencing and the NGS results. This validates the use of Sanger traces to estimate the quality of a library.

NGS resulted in 846 322 DNA reads, and over 97% of these aligned to the Cre-based index sequence using default settings in the program Bowtie2. Both the Sanger sequencing chromatograms and NGS counts show that the expected mutations and variations were very well-represented in the library extracted from the cells. The NGS resulted in at least 56 000 reads for each position within the sequence, and the fraction of each nucleotide in the mutated regions is presented in Table 2. In cases where a simple mutation was encoded, the expected mutation was present in ∼99% of the reads. When two bases were encoded, ratio pairs ranged from 62%:38% to 50%:49%. When three bases were encoded, ratio triplets ranged from 52%:29%:19% to 37%:34%:29%.

Outside of the mutated nucleotides, the average frequency of unexpected bases is 0.209% (SD = 0.093%, with an average of 210 113 reads at each position). Most of these changes are single base substitutions. The frequency of these random errors is similar within and outside of the regions covered by the mutagenic primers. For instance, the average error rate for bases within three nucleotides of a mutated base is 0.188% (SD = 0.074%). An internal PhiX control sequence with a different bar code was spiked into the library before sequencing. The average per-base error rate in the PhiX control was 0.106% (SD = 0.134%, with an average of 2018 reads at each position). Thus, errors are about 0.1% more common in the library than in the control. Interestingly, the errors in the library are seen with similar frequency within and outside of the primer-encoded regions.

We found little bias from the starting sequence at positions where the template encoded one of the bases included in that site’s base mixture. The template-encoded base was most highly represented in 46% of such cases (6 mutated positions out of 13). The fraction expected by chance is 37% (4 positions encoded 2 bases, and 9 positions encoded 3 bases, in this particular library). Thus, the 13 data points suggest a weak preference for mutagenic oligonucleotides that are more complementary to the template. Still, this preference seems to be minor, and since we do not know the precise base ratios in the mutagenic primers, it is difficult to draw any quantitative conclusions. We emphasize that the ratios we observe indicate very strong representation of all encoded nucleotides, and we expect these ratios will be sufficient for most applications.

We also observed very low levels of template sequence at positions where the template base was not included in the mutagenic primers (0.1–1.1%). Since such templated-base errors are not uniform across all sites, these imperfections in the library are unlikely to arise from a failure to fully degrade the template before library amplification. As further discussed in the Supplementary materials, it is most likely that these errors arise when one of the mutagenic primers fails to anneal to the template during the elongation/ligation step. Again, we expect that such errors will likely be acceptable for most applications. Moreover, as discussed in the Supplementary materials, these data suggest that SLUPT yields significantly lower background from the starting sequence than some alternative methods.

The high frequency of primer incorporation described above suggests that SLUPT may be useful for multisite mutagenesis applications. To validate the utility of SLUPT for this purpose, we synthesized a series of DNA mutants that included insertions, deletions and substitutions. We initially performed a series of simple mutations wherein we used SLUPT to create a mutant having a single base insertion, deletion or substitution. Primers used for all of the recombinase SLUPT studies are shown in Supplementary Table S1. As a more challenging test, we then used SLUPT to create a nine base pair deletion and nine base pair insertion. In each case the resulting mutants had no obvious background from the starting sequence (Supplementary Figure S2). To test the ability to make multiple three base pair changes across the gene, we used SLUPT to make 5 mutations simultaneously using five donor primers spaced 50 bp apart. The five donor primers aligned to the template are shown in Supplementary Figure S3. After confirming the expected mutations by Sanger sequencing of the SLUPT PCR product (data not shown), the DNA was cloned into a plasmid and transformed into *E. coli* as above. Six single colonies were sequenced in both directions. Consistent with the high rate of primer incorporation seen in our NGS experiment, all six colonies contained all five mutations. Consistent with the error rate described above, three of the six clones had point mutations outside of the mutated bases. One of these mutations, a single nucleotide deletion, was in a region covered by the mutagenic primers, but outside of the region that was mutated. The other two mutations were base substitutions that occurred in regions between primers.

We also looked to see if the spacing between the donor primers was a factor in the efficiency of the mutated products, and we found that it was not (data not shown). Primers cannot overlap, but they can be very close to one another. The closest donor primers tested to date are 2 bp apart, and the farthest primer sets tested are 440 bp apart (data not shown). We have not yet had occasion to synthesize libraries with more than six donor primers, but previous work with single-stranded DNA templates has shown that as many as 10 primers can be used in simultaneous mutagenesis reactions (18). Provided the primers do not hybridize to each other, our results suggest that using additional donor primers in reactions with PCR-derived dU-containing templates should also not be problematic.

### Antibody library generation

We speculated that SLUPT would be well suited for antibody engineering, and particularly for construction and optimization of single chain antibody (scFv) molecules. These molecules have a variety of uses in the laboratory and clinic (19, 20). scFvs can be developed *ab initio*, by screening libraries with randomized antibody fragments (21), or they can be constructed by splicing together sequences from the Fv heavy and Fv light chains of intact antibodies with the desired specificity (22, 23). In all cases, a protein linker (typically 15–20 amino acids long) is used to connect the two immunoglobulin domains. Strategies for generating site-directed scFv libraries usually rely on primer extension, gene assembly, recombination or single-stranded templates to generate the necessary genetic diversity (14, 22, 24, 25).

SLUPT allows for selective targeting of residues in the paratope that have been determined to be important to epitope binding based on structural and/or interaction studies of the antibody and ligand. To test SLUPT in the context of scFv library construction, we chose to use an antibody against cytotoxic T-lymphocyte-associated protein 4 (CTLA-4) as an example. CTLA-4 is an immune checkpoint molecule that is involved in down-regulation of the T-cell-mediated immune response (26, 27). Two monoclonal CTLA-4 antibodies have been developed and undergone clinical testing; Ipilimumab has been effective in the treatment of melanoma (28), and Tremelimumab has been used in multiple phase III clinical trials (29, 30). Crystal structures of both an scFv version of Ipilimumab and the Fab fragment of Tremelimumab have been determined in complex with CTLA-4. These structures revealed that Tremelimumab and Ipilimumab target the same epitope of CTLA-4 and have very similar structures (31, 32).

**Figure 4.**
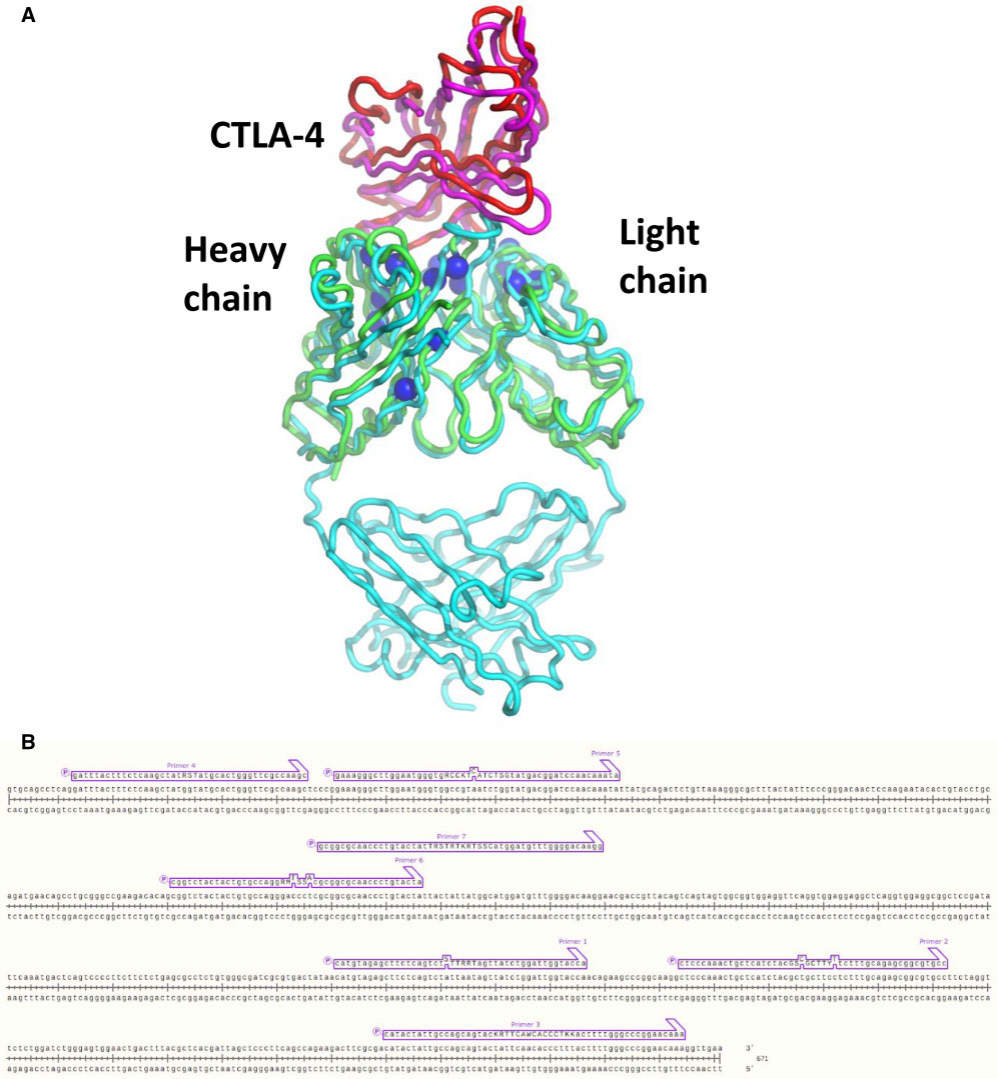
Design of library via SLUPT for anti CTLA-4 scFv antibody. (**A**) Superimposed crystal structures of the scFv ipilimumab (green, pdb code 5XJ3) and the Fab fragment of tremelimumab (teal, pdb code 5GGV). CTLA-4, seen in both structures is magenta and red, respectively. The locations of the mutated residues within the library are shown as blue spheres. (**B**) SLUPT donor primers used to create the DNA libraries of the scFv gene in a single reaction. The donor primers are in a purple box aligned to the wt scFv sequence. The overlapping primers were not used together, and there are six donor primers per library. The primers span ∼650 nucleotides, the closest pair is 5 bp, the farthest pair is ∼130 bp. The scFv PCR product is ∼900 base pairs.

To demonstrate how SLUPT might be used in a situation like this, we examined the two structures and identified residues that both interact with CTLA-4 and differ in the antibody sequences. We then generated a library consisting of a mixture of these residues (Figure 4). The library was designed using Tremelimumab as the starting gene. The seven light-chain residue positions and ten heavy chain positions are detailed in Table 3. The primers used to make these mutations and the base mixtures at the mutated positions are listed in Supplementary Figure 4b. In some instances, the genetic code made it impossible to encode only the two desired amino acids (capitalized letters in Table 3); two other codons were also encoded (lower case and gray). To avoid having to use a very long primer, libraries were generated with either primer 6 or primer 7. It was necessary to create two separate libraries because of the overlapping nature of these primers.

**Table 3.** SLUPT Library design for anti CTLA-4 scFv studies.

Primer	Chain	Amino acid variants^a^
1	Light	Position 29: L/V, position 30: N/G/s/d
2	Light	Position 50: A/G, position 52: S/F
3	Light	Position 92: Y/G/c/d, position 94: T/S, 96: F/W/c/l
4	Heavy	Position 33: G/T/a/s
5	Heavy	Position 49: A/T, position 50: V/F, position 52: W/S
6	Heavy	Position 99: D/T/a/n, position 100: P/G/r/a
7	Heavy	Position 108: Y/W/c/*, position 109: Y/C, position110: Y/G/c/d, 111: G/P/r/a

a Amino acid residues present in the two antibodies are shown in capital letters, additional mutations encoded by the base mixtures chosen are shown in lower case. The asterisk in primer 7 denotes an encoded stop codon.

In both cases, as with the earlier Cre recombinase libraries, Sanger sequencing was used to confirm the presence of the various nucleotides in the libraries (Supplementary Figure S4). The results were similar to those discussed above, with no obvious bias towards the starting sequence and good representation of all alternative bases at the selected sites of genetic variation.

### A program to assist in degenerate codon selection

To maximize the utility of SLUPT, it is important to optimize the base choices at each varied position within the synthesized libraries. A number of clever base mixtures for use in library creation have been described. The goal of these mixtures is to maximize the number of amino acids at each varied position while minimizing the redundancies that are intrinsic to the genetic code. For instance, the ‘small intelligent’ approach uses four mixtures to encode each of the 20 amino acids just once (33), and the NDT approach encodes just 12 chemically varied amino acids just once with a single mixture (34). Another productive approach has limited the size of DNA libraries by sampling only amino acids that are seen in sequence alignments of homologous proteins or based on 3D structures. In these cases, the optimal base mixtures can be chosen by entering the desired amino acids into programs such as MDC Analyzer (17, 35).

To keep things relatively simple and cost-effective, particularly in cases where a variety of amino acids are being varied via a single donor primer, we considered the complete set of possible codon mixtures that can be synthesized using a conventional DNA synthesizer. At each of the three positions within a codon, one can have 15 possibilities (1 mixture of all four bases, 4 mixtures with one base missing, 6 mixtures of two bases, and 4 individual bases). Thus, there are 15^3^ = 3375 possible choices for each codon. To assist users in selecting the most appropriate codon mixture, we have written a python script, named MSCS (Mixed Synthesis Codon Selector). This software tool takes as input the desired list of amino acids one wishes to encode and a series of weights (values between −1 and 1) that describe how important each amino acid is to the user. Negative weights are used in cases where one wishes to reduce the likelihood of seeing the specified amino acid near the top of the output list of suggested codons. Users can also input parameters that reduce the likelihood of seeing mixtures containing stop codons, mixtures that encode extra amino acids, and mixtures where requested amino acids are missing. Using this information, an ordered list of potential codon mixtures that best satisfies the request is presented to the user.

For instance, with default parameters, inputting the amino acid list A, T, F, W, C with weights 1.0, 1.0, 0.5, 1.0 and 0.2 yields the output below (only the top of the list is shown). Each line of the output is a codon mixture, and the bases within this mixture are described by the last three characters. The program uses a standard nomenclature for base mixtures (see Methods section). The top line of the output indicates that a codon with A/G/T in the first position, G/C in the second position, and G in the third position encodes each of the most desired amino acids one time, but does not encode F or C which were also requested but weighted less highly. Although they were not requested, this mixture also encodes R, S and G. Looking down the list, one sees two solutions (in bold) where all five of the requested amino acids are encoded, but not with the same frequency, and with seven unrequested amino acids also in the mix. As demonstrated here, there is not always one obviously best codon mixture. Even in these cases, however, this tool should be helpful in making informed choices as one designs a DNA library.

3/5 Encoded: A1 T1 W1 Missing: F C Extra: S1 G1 R1 Codons: 6 Code: dsg

2/5 Encoded: T1 A1 Missing: F C W Extra: 0 Codons: 2 Code: rca

2/5 Encoded: T1 A1 Missing: F C W Extra: 0 Codons: 2 Code: rct

2/5 Encoded: T1 A1 Missing: F C W Extra: 0 Codons: 2 Code: rcg

2/5 Encoded: T1 A1 Missing: F C W Extra: 0 Codons: 2 Code: rcc

3/5 Encoded: A1 T1 F1 Missing: C W Extra: S1 V1 I1 Codons: 6 Code: dyt

3/5 Encoded: A1 T1 F1 Missing: C W Extra: S1 V1 I1 Codons: 6 Code: dyc

2/5 Encoded: T1 A1 Missing: F C W Extra: S1 Codons: 3 Code: dca

2/5 Encoded: T1 A1 Missing: F C W Extra: S1 Codons: 3 Code: dct

2/5 Encoded: T1 A1 Missing: F C W Extra: S1 Codons: 3 Code: dcg

2/5 Encoded: T1 A1 Missing: F C W Extra: S1 Codons: 3 Code: dcc

2/5 Encoded: T1 A1 Missing: F C W Extra: P1 Codons: 3 Code: vca

2/5 Encoded: T1 A1 Missing: F C W Extra: P1 Codons: 3 Code: vct

2/5 Encoded: T1 A1 Missing: F C W Extra: P1 Codons: 3 Code: vcg

2/5 Encoded: T1 A1 Missing: F C W Extra: P1 Codons: 3 Code: vcc

3/5 Encoded: A1 T1 W1 Missing: F C Extra: S1 G1 R2 P1 Codons: 8 Code: nsg

**5/5 Encoded: A2 T2 F1 W1 C1 Missing:0 Extra: L1 S3 V2 G2 R1 I1 M1 Codons: 18 Code: dbk**

**5/5 Encoded: A2 T2 F1 W1 C1 Missing:0 Extra: L1 S3 V2 G2 R1 I1 M1 Codons: 18 Code: dbs**

4/5 Encoded: A2 T2 W1 C1 Missing: F Extra: S3 G2 R1 Codons: 12 Code: dsk

4/5 Encoded: A2 T2 W1 C1 Missing: F Extra: S3 G2 R1 Codons: 12 Code: dss

2/5 Encoded: T1 A1 Missing: F C W Extra: I1 V1 Codons: 4 Code: rya

2/5 Encoded: T1 A1 Missing: F C W Extra: I1 V1 Codons: 4 Code: ryt

## Discussion and conclusions

The SLUPT approach was principally inspired by three earlier methods. As mentioned earlier, in 1985, Kunkel described a multi-site mutagenesis method involving a single-stranded, phage-derived dU-containing template (15). In 2003, Coco and coworkers described a gene shuffling strategy termed RACHITT, which utilized a single-stranded dU-containing template to direct the assembly of related gene fragments (36), and in 2004, Seyfang and Jin described a multi-site mutagenesis method using a single-stranded conventional DNA template (18). The first method was somewhat cumbersome because it requires a special strain of bacteria, phage infection and phage DNA isolation prior to library synthesis. The second method does not work with synthetic donor primers because the 5′ exonuclease activity of the polymerase used degrades the primers. The third method differs significantly from SLUPT and the other two in that it does not involve degradation of the nontemplate strand or inactivation of the template after the product strand has been synthesized. Thus, relative to earlier methods, SLUPT simplifies the process of generating the template, eliminates the heating step prior to primer annealing (likely leading to more uniform sampling) and utilizes a high fidelity polymerase that is compatible with both dU bases and relatively short mutagenic primers.

Many protein engineering projects proceed in two phases. Initially, targeted mutations may be made in rationally chosen regions (i.e. CDR loops of antibodies or regions nearby the active site of an enzyme). Once the desired activity has been detected, random mutagenesis is often used as a second step, to identify changes that optimize properties such as solubility, stability, binding and/or enzymatic activity. The comparatively uniform sampling of mutations in selected regions makes SLUPT ideally suited for the initial screening phase of the protein engineering workflow. Furthermore, the speed and minimal cost associated with synthesis of subsequent libraries, along with the ease with which random mutations can be incorporated alongside the targeted changes (i.e. by using error-prone PCR in the final amplification step), suggests that SLUPT may facilitate improvements to protein engineering workflows. For example, one might envision scenarios where a large series of different libraries, each with diversity in multiple, different noncontiguous protein domains, are screened so as to narrow down the regions that are truly most important for altered function. One might also envision the synthesis of second generation libraries with expanded diversity in these key regions. Finally, one might envision using the results from these initial cycles of selection to refine, but not completely restrict diversity in key regions at the same time that random mutations are introduced as is common in OSCARR (14).

As noted earlier, a variety of approaches can be used to simultaneously mutate multiple regions of a gene. In its current form, SLUPT is best suited for cases where the region of DNA that will be mutated is <3 kb. This is because PCR synthesis of dU-containing DNA is less efficient than conventional PCR, and the yield becomes an issue as the products get longer. Although we have synthesized and purified single stranded dU-containing templates as long as 5 Kb (data not shown), to date we have not had occasion to generate SLUPT libraries with such long templates. Earlier work with dU-containing single-stranded phage-derived templates (18) indicates that plasmid-length templates should not be problematic. It is important to note that SLUPT is best suited for cases where a linear PCR fragment is an acceptable product from the library synthesis. Linear DNA can be used in ribosome display and related techniques (37). Linear DNA is also common in protein engineering workflows that rely on PCR mutagenesis of the gene in question but not the surrounding vector. It is notable that the output of some other procedures, including gene assembly and multi-site Quikchange, is a plasmid, not a linear DNA fragment. In deciding whether to use SLUPT, it is also important to consider the random error rate, about 1 in 1000 bases. With shorter genes this error rate may be acceptable, and in some screening projects it may even be desirable. However, with longer genes, the frequency of random errors will become problematic (see Supplementary discussion).

Finally, we emphasize that while preparation of the single-stranded, dU-containing template involves more effort relative to some other approaches, a single template preparation is sufficient for synthesis of numerous subsequent libraries and/or mutations. As a consequence, SLUPT is particularly well suited for situations where rapid, inexpensive synthesis of a series of libraries or mutations is beneficial. We anticipate that SLUPT will find application in a broad array of directed evolution, mutagenesis and protein engineering efforts. It will allow users to better use sequence alignments and structural information to enhance the rate at which desirable mutations are uncovered. The speed, efficiency, low cost and robustness of this approach, along with the stoichiometrically balanced nature of the product libraries, make SLUPT well suited for many applications. 

## SUPPLEMENTARY DATA

Supplementary Data are available at SYNBIO Online.

## Funding

This study was funded by National Institutes of Health grant R01GM126149. 

*Conflict of interest statement*. None declared. 

## Supplementary Material

ysaa030_Supplementary_Data
