# Supplementary material for: Synthesis of libraries and multi-site mutagenesis using a PCR-derived, dU-containing template
Source: Synth Biol (Oxf). 2021 Jan 5;6(1):ysaa030. doi: 10.1093/synbio/ysaa030 (PMC8260824; doi:10.1093/synbio/ysaa030)
Supplement: ysaa030_Supplementary_Data [file ysaa030_supplementary_data.zip › supplementary_figures.pdf]

Supplemental Figure S1.

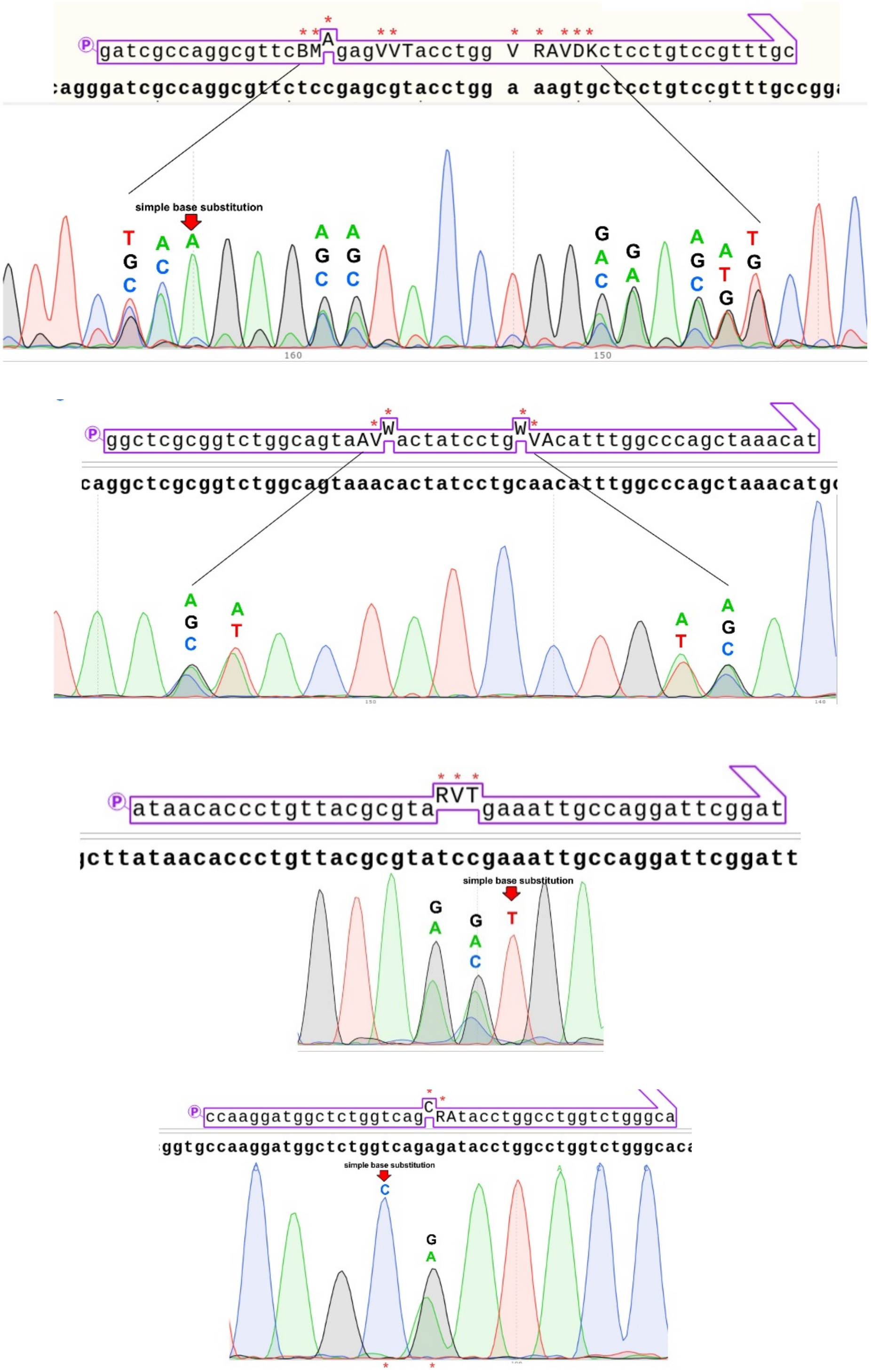

Figure S1. Sanger sequencing results from a recombinase library that was generated using four mutagenic donor primers. In each case, the 5' phosphorylated donor primer (purple box) is shown aligned the starting sequence (lowercase bold). Below is the interpreted sequence and the Sanger sequencing trace. As discussed in the main text, this same library was also subjected to NexGen sequencing.

Supplemental Figure S2.

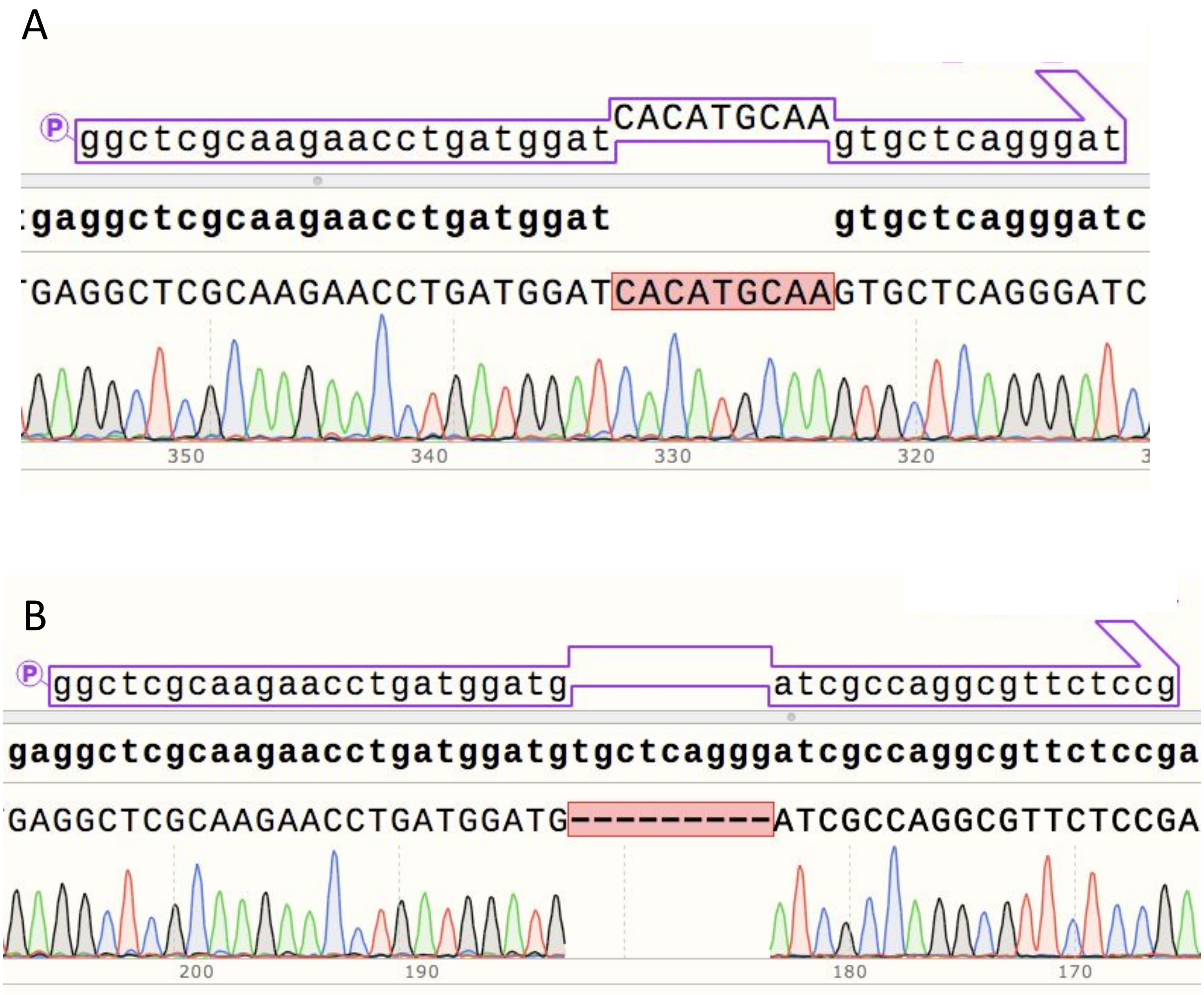

Figure S2. Sanger sequencing results of recombinase mutants containing a nine base pair insertion (A) or deletion (B) that were created using the SLUPT method. The 5' phosphorylated donor primer (purple box) is shown aligned the starting sequence (lowercase bold). Below the starting sequence is the interpreted sequence and the Sanger sequencing trace. These traces demonstrate that the mutagenesis has been successful in a large majority of DNA molecules.

Supplemental Figure S3.

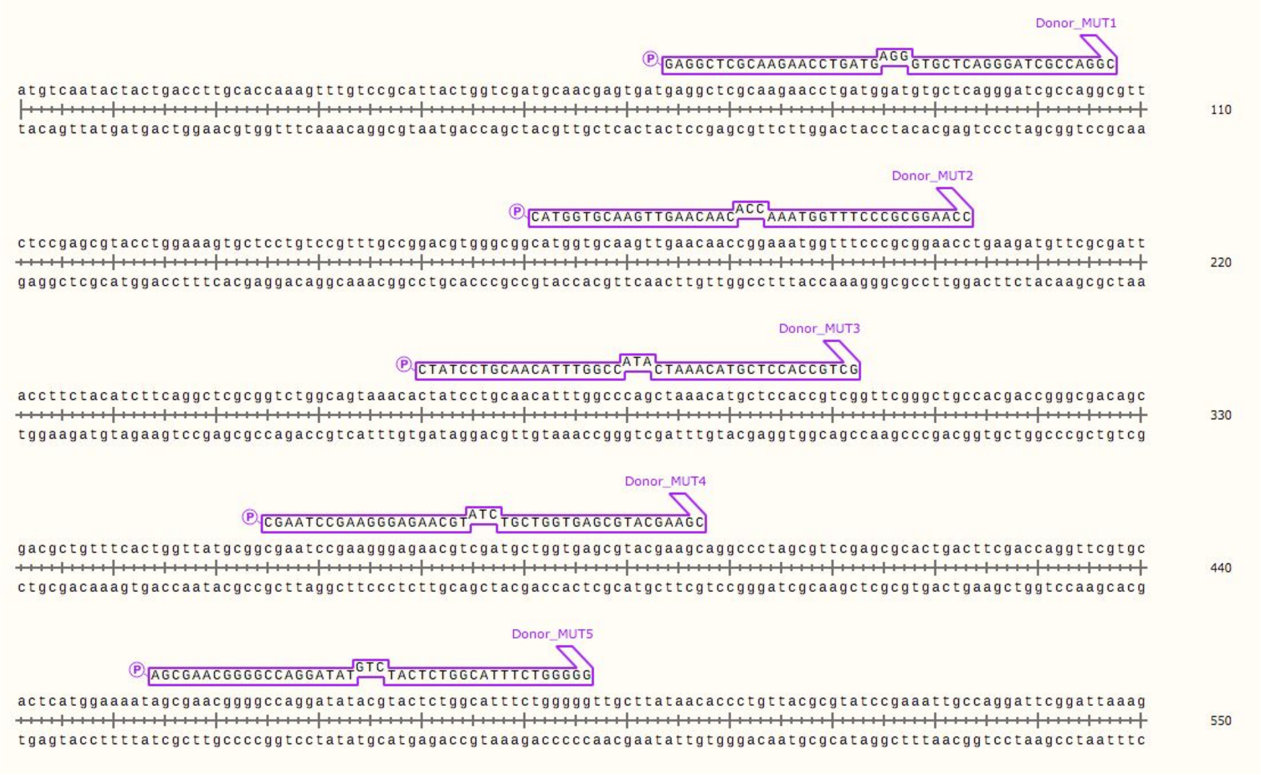

Figure S3. Five SLUPT primers spaced across recombinase gene. This figure shows the donor (outlined in purple box) aligned to the recombinase template. The primers are each separated by ~50 bases and were used simultaneously in SLUPT for mutagenesis spread across a gene. Each donor primer changes 3 bases.

Supplementary Figure S4

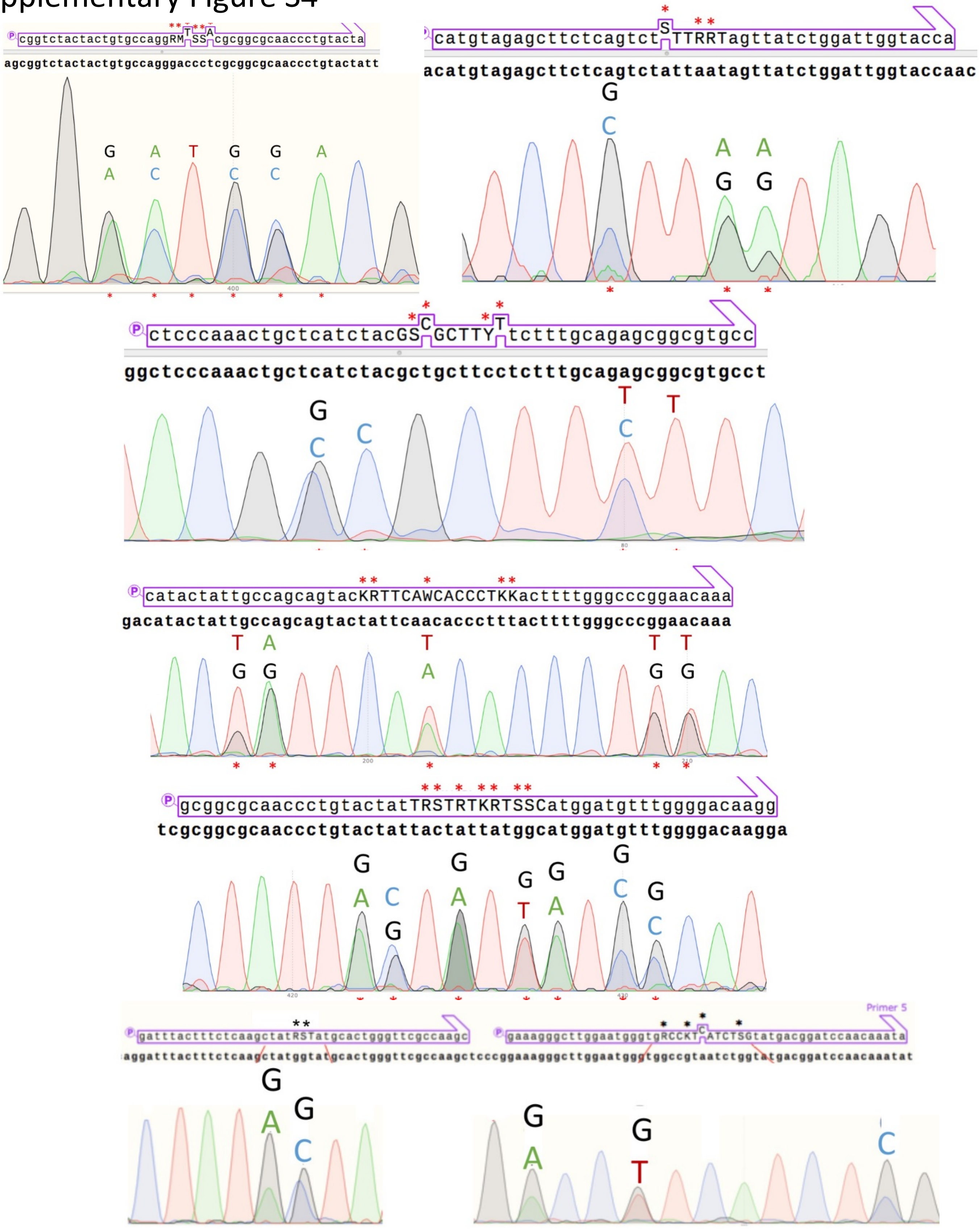

Figure S4. Sanger sequencing of seven mutated anti CTL-4 antibody regions. This figure shows the donor primers (outlined in purple box) aligned to the scFv template. Altered bases are indicated by a *.
