# Supplementary material for: Synthesis of libraries and multi-site mutagenesis using a PCR-derived, dU-containing template
Source: Synth Biol (Oxf). 2021 Jan 5;6(1):ysaa030. doi: 10.1093/synbio/ysaa030 (PMC8260824; doi:10.1093/synbio/ysaa030)
Supplement: ysaa030_Supplementary_Data [file ysaa030_supplementary_data.zip › Table donor primers 11-08-2020.docx]

Supplemental Table S1. Mutagenic Primers used for variant library and mutagenesis via SLUPT

| **Donor Primer**  **Name** | **5’🡪3’ Donor primer sequences**  **(all are 5’ phosphorylated)** | **N** | **5’** | **3’** |
| --- | --- | --- | --- | --- |
| Cre Library 1 |  |  |  |  |
| helixB | CGTTCTCCGAGCGTACCTGG**RRK**GTGCTCCTGTCCGTTTGCCG | 43 | 20 | 20 |
| helixD | GTCTGGCAGTAAACACTATC**VNAVN**ACATTTG**DS**C**VDS**CTAAACATGCTCCACCGTCG | 58 | 20 | 20 |
| helixD  (shorter) | GCAGTAAACACTATC**VNAVN**ACATTTG**DS**C**VDS**CTAAACATGCTCCAC | 48 | 15 | 15 |
| Cre Library 2 |  |  |  |  |
| region 1 | GATCGCCAGGCGTTC**BMA**GAG**VV**TACCTGG**VR**A**VDK**CTCCTGTCCGTTTGC | 51 | 15 | 15 |
| region 2 | GGCTCGCGGTCTGGCAGTAA**VW**ACTATCCTG**WV**ACATTTGGCCCAGCTAAACAT | 54 | 20 | 21 |
| region 3 | ATAACACCCTGTTACGCGTA**RVT**GAAATTGCCAGGATTCGGAT | 43 | 20 | 20 |
| region 4 | CCAAGGATGGCTCTGGTCAG**CR**ATACCTGGCCTGGTCTGGGCA | 43 | 20 | 21 |
| Mutations |  |  |  |  |
| 1 bp change | CCTGATGGAT**A**TGCTCAGGGATCGCCAGG | 29 | 10 | 18 |
| 1 bp deletion | CTCGCAAGAACCTGATG**-**ATGTGCTCAGGGATCGCC | 35 | 17 | 18 |
| 1 bp insertion | CTCGCAAGAACCTGATGGAT**C**GTGCTCAGGGATCGCC | 37 | 20 | 16 |
| 9 bp insertion | ggctcgcaagaacctgatggat**CACATGCAA**gtgctcagggat | 43 | 22 | 12 |
| 9 bp deletion | ggctcgcaagaacctgatggatg**_________**atcgccaggcgttctccg | 41 | 23 | 18 |
| 3 bp change | GAGGCTCGCAAGAACCTGATG**AGG**GTGCTCAGGGATCGCCAGGC | 44 | 21 | 20 |
| 3 bp change | CATGGTGCAAGTTGAACAAC**ACC**AAATGGTTTCCCGCGGAACC | 43 | 20 | 20 |
| 3 bp change | CTATCCTGCAACATTTGGCC**ATA**CTAAACATGCTCCACCGTCG | 43 | 20 | 20 |
| 3 bp change | CGAATCCGAAGGGAGAACGT**ATC**TGCTGGTGAGCGTACGAAGC | 43 | 20 | 20 |
| 3 bp change | AGCGAACGGGGCCAGGATAT**GTC**TACTCTGGCATTTCTGGGGG | 43 | 20 | 20 |
| 3 bp change | ctccgagcgtacctggaaa**act**ctcctgtccgttt | 35 | 19 | 13 |
| 3 bp change | cgtacctggaaagtgctcct**ACG**cgtttgccggacgtgggcgg | 43 | 20 | 20 |
| Antibody libraries |  |  |  |  |
| Primer 1 | catgtagagcttctcagtct***STTRRT***agttatctggattggtacca | 46 | 20 | 20 |
| primer 2 | ctcccaaactgctcatctac***GSC***gct***TYT***tctttgcagagcggcgtgcc | 49 | 20 | 20 |
| primer 3 | catactattgccagcagtac***KRT***tca***WCA***ccc***TKK***acttttgggcccggaacaaa | 65 | 20 | 20 |
| primer 4 | gatttactttctcaagctat***RST***atgcactgggttcgccaagc | 43 | 20 | 20 |
| primer 5 | gaaagggcttggaatgggtg***RCCKTC***atc***TSG***tatgacggatccaacaaata | 62 | 20 | 20 |
| primer 6 | cggtctactactgtgccagg***RMTSSA***cgcggcgcaaccctgtacta | 46 | 20 | 20 |
| primer 7 | gcggcgcaaccctgtactat***TRSTRTKRTSSC***atggatgtttggggacaagg | 52 | 20 | 20 |

Red indicates the mutated region. Single letter codes are used to denote base mixtures. N is the length of the primer, 5’ is the number of bases on the 5’ side of the mutation, 3’ is the number of bases on the 3’ side of the mutation.
